# Intravitreal triamcinolone for cancer-associated retinopathy refractory to systemic therapy

**DOI:** 10.1007/s12348-012-0067-9

**Published:** 2012-03-14

**Authors:** Nancy Huynh, Yevgeniy Shildkrot, Ann-Marie Lobo, Lucia Sobrin

**Affiliations:** Massachusetts Eye and Ear Infirmary, Department of Ophthalmology, Harvard Medical School, 243 Charles Street, Boston, MA 02114 USA

**Keywords:** Cancer-associated retinopathy, Triamcinolone, Steroids, Intravitreal injection

## Abstract

**Purpose:**

The purpose of this study is to report the use of intravitreal triamcinolone for treatment of cancer-associated retinopathy (CAR) refractory to systemic therapy.

**Methods:**

This was a retrospective chart review study.

**Results:**

A 67-year-old man presented with cancer-associated retinopathy with antibodies against a 46-kDa retinal protein, alpha enolase. There was disease progression despite therapy with mycophenolate and intravenous immunoglobulin. Serial intravitreal injections of triamcinolone resulted in restoration of photoreceptor anatomy on optical coherence tomography and visual improvement. The patient’s vision was preserved at 20/40 OD and 20/32 OS until his death from lung cancer 31 months after CAR diagnosis.

**Conclusions:**

Intravitreal triamcinolone may be beneficial for maintenance of vision in patients with CAR.

## Introduction

Cancer-associated retinopathy (CAR) is a paraneoplastic process caused by autoantibodies against retinal proteins, including recoverin and alpha enolase [1, 2]. Approximately 50 % of patients with CAR present with visual symptoms before the diagnosis of a malignancy is made [3]. CAR is rare, and there are currently no standard treatment regimens for the condition [4]. Systemic corticosteroids, steroid-sparing immunosuppression, and non-traditional immunomodulatory therapies including intravenous immunoglobulin (IVIg) have been shown to variably preserve or improve vision [5–7]. Cystoid macular edema (CME) has been reported in patients with non-paraneoplastic autoimmune retinopathy (npAIR) but is less common with CAR [2]. We report a patient with CAR in whom serial intravitreal steroid injections resulted in maintenance of visual function for more than 2 years until his death.

## Case report

**Fig. 1:**
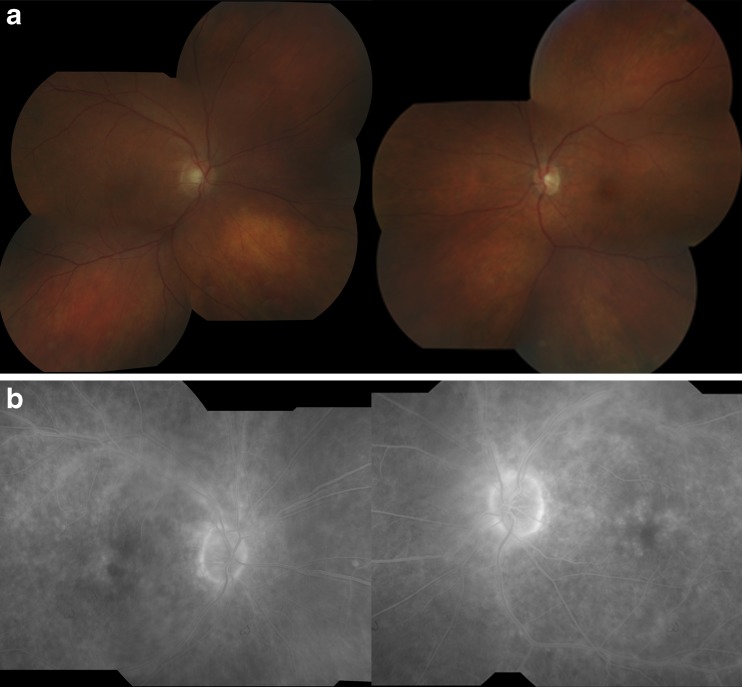
**a** Fundus photos showing macular edema and arteriolar narrowing. **b** Fluorescein angiography showing petalloid leakage in the macula, perivascular leakage, and disk staining

A 67-year-old man presented with decreased vision, photopsias, and nyctalopia. Examination revealed best corrected visual acuities of 20/32 in the right eye and 20/25 in the left eye. Dilated fundus exam was remarkable for bilateral CME and arteriolar narrowing (Fig. 1a). Fluorescein angiography (FA) revealed petalloid leakage at the fovea, perivascular leakage, and disk staining bilaterally (Fig. 1b). Multifocal and full-field electroretinography (ERG) revealed severely decreased scotopic and photopic responses but no negative ERG pattern. Serologic testing revealed the presence of antibodies to a 46-kDa retinal protein that was confirmed to be alpha enolase. Systemic evaluation for malignancy uncovered poorly differentiated squamous cell carcinoma of the lung. Chemotherapy was initiated with carboplatin and paclitaxel. For his eye disease, the patient was initially treated with intravenous methylprednisolone, 1 g/day for 3 days, followed by oral prednisone (initial dose of 80 mg/day and then a taper over 2 months) with decreased retinal vascular leakage and CME.

**Fig. 2:**
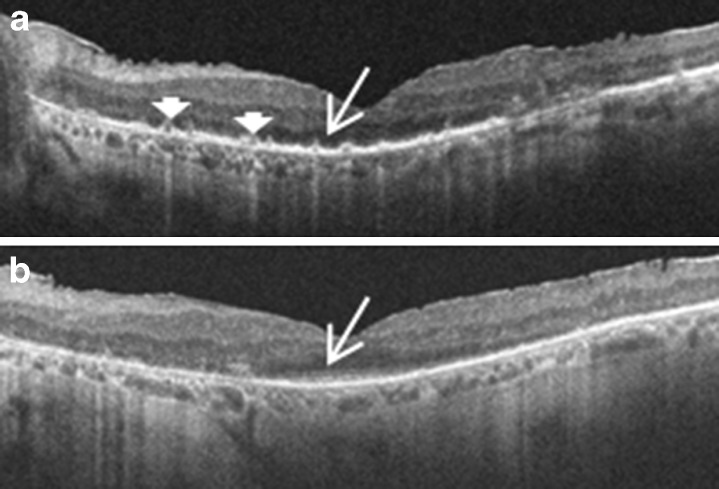
**a** OCT of left eye showing loss of the inner segment/outer segment (IS/OS) junction subfoveally (*arrow*) and hyperreflective areas along the photoreceptor layer (*arrowheads*), possibly indicative of foci of active inflammation. The patient’s vision was hand motions. **b** OCT 1 month after intravitreal steroid injection. The IS/OS junction has been restored subfoveally (*arrow*) and the hyperreflective areas have largely resolved, with vision improving to 20/32

The patient’s visual acuities worsened to 20/125 OD and 20/60 OS with tapering of the prednisone, and FA showed worsening retinal vasculitis. Steroid-sparing treatment was attempted first with mycophenolate mofetil (2 g/day) and then with four doses of IVIg, with no response to either therapy. FA continued to show CME and diffuse retinal vasculitis. Intravitreal triamcinolone (IVTA) was administered bilaterally, with vision improving to 20/40 OD and 20/32 OS and resolution of cystic changes. Over the ensuing 2.5 years, the patient received four additional IVTA injections bilaterally. When the IVTA effect waned, his vision diminished to as low as 20/70 in the right eye and hand motions in the left eye. After the initial injection, there were no retinal cystic changes or significant retinal thickening on optical coherence tomography (OCT) at the subsequent visits, yet the patient periodically reported visual deterioration and had objectively diminished visual acuity with disruption of photoreceptor anatomy on OCT (Fig. 2a). The vision and anatomical changes improved with IVTA (Fig. 2b). His visual acuities were 20/40 OD and 20/32 OS after the most recent IVTA injections. The patient developed steroid response intraocular pressure rises in both eyes that decreased with topical antihypertensive therapy. No other complications of IVTA injections occurred. He passed away from complications of his lung cancer 31 months after his CAR diagnosis.

## Discussion

This report describes the successful treatment of CAR with intravitreal steroids. The treatment of CAR is often challenging. Treatment of the underlying malignancy alone does not alter the course of the ocular disease. Various treatment modalities have been tried in patients with CAR, including oral and intravenous steroids, plasmapheresis, IVIg, rituximab, azathioprine, cyclosporine, and mycophenolate mofetil [2, 6–8]. Despite treatment with these systemic medications, it is not unusual to have a progressive decline in vision with this disease. Our patient responded initially to systemic steroids but did not have an alteration of his disease course with mycophenolate mofetil or IVIg. After initiation of IVTA injections, his vision improved. Vision improvement in CAR is unusual; vision stabilization is often the best that can be hoped for. In this patient, there was strong evidence for a causal relationship between the injections and visual improvement. When the intravitreal triamcinolone wore off, his vision declined. With his initial injections, the improvement in vision could be attributed, at least in part, to treatment of the CME. But subsequent injections were given when there were no retinal cystic changes or thickening present on OCT and yet reinjection led to marked subjective and objective improvement. The improvement was likely due to suppression of inflammation around the photoreceptors as evidenced by restoration of inner segment/outer segment junction and resolution of hyperreflective lesions along the photoreceptor layer on OCT after the injections. In one such instance, his vision recovered from hand motions to 20/32 OS.

CME and periphlebitis can be seen in patients with npAIR but is less common with CAR. There is one case report of retinal periphlebitis associated with CAR in a patient with ovarian cancer [9]. In the presence of CME, intravitreal steroids have previously been shown to be effective in patients with npAIR [2]. The unique presentation of CAR with CME and vasculitis in our patient motivated the use of intraocular triamcinolone, but there was evidence for an effect on the course of CAR directly given the visual improvements even when IVTA injected in the absence of CME. The presence of anti-retinal antibodies in the sera of patients with CAR suggests the need for systemic intervention in general, but this patient’s course suggests that a subset of CAR patients might be successfully treated with local therapy. The OCT photoreceptor findings in this patient pre- and post-injection indicate that intravitreal steroids may be able to successfully suppress inflammation and apoptosis induced by the autoantibodies at the level of the photoreceptors despite ongoing systemic production and/or circulation of antibodies. Some authors caution that readministration of intravitreal steroid for recurrent CME in npAIR and CAR is best done early when cystic changes are just starting to recur to avoid cycles of retinal cyst expansion and collapse which may cause retinal damage [2, 10]. Similarly, CAR patients without CME being treated with intravitreal steroids should be followed closely with OCT imaging and retreated promptly when evidence of photoreceptor layer disruption becomes apparent to avoid permanent photoreceptor damage.

In summary, intravitreal steroids should be considered in patients with CAR who do not respond to systemic therapy. In our patient, serial IVTA injections led to preservation of vision for almost 3 years.

## References

[1] Adamus G, Ren G, Weleber RG (2004). Autoantibodies against retinal proteins in paraneoplastic and autoimmune retinopathy. BMC Ophthalmol.

[2] Ferreyra HA, Jayasundera T, Khan NW, He S, Lu Y, Heckenlively JR (2009). Management of autoimmune retinopathies with immunosuppression. Arch Ophthalmol.

[3] Khan N, Huang JJ, Foster CS (2006). Cancer associated retinopathy (CAR): an autoimmune-mediated paraneoplastic syndrome. Semin Ophthalmol.

[4] Chan JW (2003). Paraneoplastic retinopathies and optic neuropathies. Surv Ophthalmol.

[5] Kashiwabara K, Nakamura H, Kishi K, Yagyu H, Sarashina G, Kobayashi K, Matsuoka T (1999). Cancer-associated retinopathy during treatment for small-cell lung carcinoma. Intern Med.

[6] Guy J, Aptsiauri N (1999). Treatment of paraneoplastic visual loss with intravenous immunoglobulin. Arch Ophthalmol.

[7] Shildkrot Y, Sobrin L, Gragoudas ES (2011). Cancer-associated retinopathy: update on pathogenesis and therapy. Sem Ophthalmol.

[8] Mahdi N, Faia LJ, Goodwin J, Nussenblatt RB, Sen HN (2010). A case of autoimmune retinopathy associated with thyroid carcinoma. Ocul Immunol Inflamm.

[9] Kim SJ, Toma HS, Thirkill CE, Dunn JP (2010). Cancer-associated retinopathy with retinal periphlebitis in a patient with ovarian cancer. Ocul Immunol Inflamm.

[10] Jr H, Ferreyra HA (2008). Autoimmune retinopathy: a review and summary. Semin Immunopathol.

